# Determination of Zinc, Cadmium, Lead, Copper and Silver Using a Carbon Paste Electrode and a Screen Printed Electrode Modified with Chromium(III) Oxide

**DOI:** 10.3390/s17081832

**Published:** 2017-08-09

**Authors:** Zuzana Koudelkova, Tomas Syrovy, Pavlina Ambrozova, Zdenek Moravec, Lubomir Kubac, David Hynek, Lukas Richtera, Vojtech Adam

**Affiliations:** 1 Department of Chemistry and Biochemistry, Mendel University in Brno, Zemedelska 1, Brno CZ-613 00, Czech Republic; alalligazza@gmail.com (Z.K.); d.hynek@email.cz (D.H.); oliver@centrum.cz (L.R.); 2 Department of Graphic Arts and Photophysics, University of Pardubice Doubravice 41, Pardubice CZ–533 53, Czech Republic; tomas.syrovy@upce.cz; 3 Center of Materials and Nanotechnologies, Faculty of Chemical Technology, University of Pardubice, Cs. Legii square 565, Pardubice CZ–53002, Czech Republic; lubomir.kubac@cocltd.cz; 4 Department of Geology and Pedology, Mendel University in Brno, Zemedelska 1, Brno CZ–613 00, Czech Republic; pavlina.ambrozova@mendelu.cz; 5 Department of Chemistry, Masaryk University, Kotlarska 2, Brno CZ–61137, Czech Republic; hugo@chemi.muni.cz; 6 Centre for Organic Chemistry Ltd., Rybitvi 296, Rybitvi CZ–533 54, Czech Republic; 7 Central European Institute of Technology, Brno University of Technology, Purkynova 123, Brno CZ-612 00, Czech Republic

**Keywords:** carbon paste, chromium, electrochemistry, heavy metals, screen-printed electrode, silver

## Abstract

In this study, the preparation and electrochemical application of a chromium(III) oxide modified carbon paste electrode (Cr-CPE) and a screen printed electrode (SPE), made from the same material and optimized for the simple, cheap and sensitive simultaneous determination of zinc, cadmium, lead, copper and the detection of silver ions, is described. The limits of detection and quantification were 25 and 80 µg·L^−1^ for Zn(II), 3 and 10 µg·L^−1^ for Cd(II), 3 and 10 µg·L^−1^ for Pb(II), 3 and 10 µg·L^−1^ for Cu(II), and 3 and 10 µg·L^−1^ for Ag(I), respectively. Furthermore, this promising modification was transferred to the screen-printed electrode. The limits of detection for the simultaneous determination of zinc, cadmium, copper and lead on the screen printed electrodes were found to be 350 µg·L^−1^ for Zn(II), 25 µg·L^−1^ for Cd(II), 3 µg·L^−1^ for Pb(II) and 3 µg·L^−1^ for Cu(II). Practical usability for the simultaneous detection of these heavy metal ions by the Cr-CPE was also demonstrated in the analyses of wastewaters.

## 1. Introduction

Toxic metals such as lead and cadmium are hazardous environmental pollutants which tend to bioaccumulate [1,2,3,4] resulting in various pathologies including cancer [5,6,7]. These are toxic to vertebrates and invertebrates even at lower concentrations [8,9]. Zinc and copper are essential elements necessary for most organisms but overexposure to these elements can also be life threatening [9,10]. Therefore, it is not surprising that levels of metals both toxic and essential have to be monitored [1,3]. Electrochemical methods or atomic absorption spectrometry (AAS) are often used for the determination of metals in cells, body liquids and/or tissues, as well as in environmental samples. AAS is a very sensitive method; however, it has many disadvantages including cost and inability to be miniaturized. In contrast, electrochemical methods are low cost and can be applied in situ [1,3,11,12,13]. The gold standard of electrochemistry is to perform a measurement with a mercury drop electrode. During the last decades, any handling of mercury was subjected to strict rules because of its high toxicity. For that reason, sensitive and suitable alternatives have been sought [13]. One of the recent trends is the production and application of variously modified carbon paste electrodes (CPEs) [3,14]. CPEs have a wide range of benefits as they are easy to use and prepare with high reproducibility and can be easily modified. Carbon-based materials are often used for the preparation of paste electrodes because of several advantages, including binding to other substances, good conductivity and their ability to form a relatively homogeneous electrode. The benefits of carbon pastes include their non-toxicity, environmental friendliness and their large electrochemical potential covering numerous applications [3,15]. Last but not least, there is also the possibility of subsequent transfer into the screen printed electrodes (SPEs) enabling low cost analyses [15]. Moreover, their production is relatively inexpensive, especially when using a PET substrate as carrier foil. They also show good reproducibility of measurements, and the foil SPE is portable, has a high sensitivity and is relatively easy to dispose of. In combination with a portable potentiostat, it allows for easy in situ measurements [2,16,17,18]. This shows that SPEs provide very good alternatives to other types of working electrodes including mercury ones.

Cr-based material was successfully approved in our previous study for simultaneous determination of Cr(III) and Cr(VI), and therefore further use of similar insoluble Cr-based material for another heavy metal detection was investigated [19]. The aim of this study was to design a suitably modified SPE that could be used to detect as many metals as possible in one step—using one electrolyte, and one method or setting ideally. It was found that the ability to detect said metals at very low concentrations is determined not only by the chemical nature of the modifier but also by its shape and active surface. Large-surface modified Cr_2_O_3_ electrodes were used for this study. It was observed that this type of modification had a very high sensitivity to silver ions, when Cr-CPE was able to detect Ag(I) down to the microgram per L. A modification with bismuth, which has similar characteristics to the mercury electrode, was used for detection of heavy metals recently [20,21,22]. The advantages of bismuth modification include a very high sensitivity, as the modified SPE achieves detection limits of the order of ng·L^−1^. However, their weakness is the inability to detect Cu(II) ions [23]. Besides the detection of one metal, the simultaneous detection of metal ions is also a central interest for numerous researchers. The simultaneous detection of Zn(II), Cd(II), Pb(II) and Cu(II) ions is often performed using a mercury electrode and/or their amalgams [24,25,26]. Their sensitivity for for Zn(II), Cd(II), Pb(II) and Cu(II) is very low, with detection limits down to µg·L^−1^ or even ng·L^−1^ [20,21,22,23,27,28,29], but these again suffer from mercury toxicity. However, simultaneous detection of Zn(II), Cd(II), Pb(II) and Cu(II) on the CPE and/or the SPE is not too common.

The aim of this study was to modify the CPE with chromium(III) oxide and to use such an electrode for the simultaneous detection of zinc, cadmium, lead and copper ions. Moreover, we used the fabricated electrode to detect Ag(I). In these cases, we tested various conditions to find the optimal ones and tested several interferences to show the ability of an electrode to be used for environmental purposes.

## 2. Materials and Methods

### 2.1. Chemicals

Chemicals ((NH_4_)_2_Cr_2_O_7_, Cr_2_O_3_, AgNO_3_, Zn(NO_3_)_2_, α-terpineol, ethyl cellulose and others) were obtained from Sigma-Aldrich (Saint Louis, MO, USA) unless noted otherwise. Expanded graphite EPGM for preparation of the CPE and SPE were purchased from Graphite Tyn Ltd. (Tyn nad Vltavou, Czech Republic).

In the study, high-purity deionized water (Milli-Q Millipore 18.2 MΩ/cm, Bedford, MA, USA) was used. Chromium(III) oxide for modification of the paste electrode was prepared by the thermal decomposition of ammonium dichromate. The prepared chromium(III) oxide was washed 9 times with water to prevent impurities caused mainly by trace contamination or residual dichromate.

### 2.2. Scanning Electron Microscopy (SEM) Analysis

The morphology of the commercially obtained chromium(III) oxide prepared by thermal decomposition was compared using the scanning electron microscope MIRA3 LMU (Tescan, a.s., Brno, Czech Republic). An accelerating voltage of 15 kV and a beam current of approximate 1 nA were used for visualization with satisfactory results regarding its maximum throughput.

### 2.3. Porosity Determination

Nitrogen adsorption/desorption experiments were performed at 77 K on a Quantachrome Autosorb-1MP porosimeter (Quantachrome GmbH & Co. KG, Odelzhausen, Germany). Surface areas (SA) and total pore volumes (*V_tot_* at *p*/p_0_ = 0.97) were determined by the volumetric technique [30]. Prior to the measurements, the samples were degassed at 20 °C for at least 20 h until the outgas rate was less than 0.4 Pa·min^−1^. The adsorption-desorption isotherms were measured for each sample at least three times. The specific surface area was determined by the multipoint BET method with eleven data points with relative pressures between 0.02 and 0.30.

### 2.4. Cr-CPE Preparation

For the preparation of the paste electrode, 100 mg of expanded graphite and 25 mg of the prepared chromium(III) oxide were mixed with 300 µL of paraffin oil. This mixture was homogenized in a mortar for 25 min and subsequently transferred by spatula into the Teflon electrode body with inner diameter of 2.5 mm.

### 2.5. Ink Formulation Preparation

The ink formulation for the screen-printing technique was made from a solution consisting of ethyl cellulose (EC) as a binder and α-terpineol as a solvent. 4 g of EC was dissolved in 96 g α-Terpineol, then stirred at 50 °C and 400 rpm for 120 min using the magnetic stirrer IKA RCT basic (IKA, Staufen im Breisgau, Germany). The expanded graphite was further disintegrated using an agate mortar and pestle. The ink formulation for the counter electrode and reference electrode (CE/RE) was prepared by incorporating the expanded graphite into EC solution with the weight ratio of EC and expanded graphite of 1:4. The expanded graphite was added to the EC solution while stirring using a magnetic stirrer. The ink formulation was mixed for a further 24 h. The WE (working electrode) ink formulation was prepared under the same procedure as the CE/RE ink formulation with the addition of the prepared chromium(III), where the final weight ratio of EC : expanded graphite : chromium(III) oxide was 1:4:1.

### 2.6. Production of the SPE

The SPE were fabricated from PET substrate (175 μm thick DuPont Teijin Films, Melinex ST504, Cleveland, UK). The layout of the printed panel of sensors consisted of 33 sensors in three rows with eleven sensors in each row. All layers were printed out using the screen-printing machine EKRA E1. The drying of selected layers was performed in the hot air oven Memmert UN55 at 120 °C for 30 min with the exception of the UV curable dielectric layer CSP-5210, where the radiation dose under a medium pressure mercury lamp was set to 600 mJ·cm^−2^. Printing stencils were created for all layers based on Saati PES mesh with 120 threads per cm coated by Dirasol 915 diazo photopolymer emulsion.

The silver current collectors were fabricated using silver conductive composite paste Dupont 5029 and were printed as first layers. In the next step, the CE and RE electrode were printed using the CE/WE ink formulation before printing the WE electrode. The last mask layer, which determines the active area of sensors, was printed using the UV curable ink formulation CSP-5210.

### 2.7. Electrochemical Detection

The electrochemical detection of Zn(II), Cd(II), Pb(II), Cu(II) and Ag(I) ions was carried out using a three electrode system connected with the 663 VA Stand (Metrohm, Herisau, Switzerland). Software NOVA 1.8 (Metrohm, Herisau, Switzerland) was used for data evaluation. As a reference electrode, Ag/AgCl (3 M KCl) was used; as a counter electrode, platinum was used and as a working electrode a Cr-CPE electrode was used. Prior to each measurement, approximately 0.1 mm of paste from the carbon paste electrode was wiped on a filter paper to obtain a new surface. Square wave anodic stripping voltammetry (SWASV) was performed in the presence of 2 M of acetate buffer, pH 5. The dosage was 3.7 mL of the sample and 300 µL of the buffer. The parameters of the SWASV measurement were as follows: an initial potential of −1.3 V, an end potential of +0.5 V, a deposition potential of −1.3 V, an accumulation time of 100 s, a voltage step of 5 mV, a pulse amplitude of 150 mV, a frequency of 150 Hz and an equilibration time of 5 s. Each result was expressed as the average of 5 measurements. The SPEs were measured with the same parameter setting and in the same buffer.

Cyclic voltammetry (CV) was measured in the same buffer with the following parameters: start potential −1.3 V, upper vertex potential 1.0 V, lower vertex potential −1.3 V, stop potential −1.3 V, number of stop crossings 8, step potential 5 mV and a scan rate of 0.75 V·s^−1^.

### 2.8. Atomic Absorption Spectrometry

Measurements were carried out using a 240 FS AA Agilent Technologies flame atomic absorption spectrometer with deuterium lamp background correction, or a 280Z Agilent Technologies atomic absorption spectrometer with electrothermal atomization and Zeeman background correction, both purchased from Agilent Technologies (Santa Clara, CA, USA). Zinc, cadmium, lead and copper were detected on the following primary wavelengths: Zn(II) 213.9 nm (spectral bandwidth 1.0 nm, lamp current 5 mA); Cd(II) 228.8 nm (spectral bandwidth 0.5 nm, lamp current 4 mA); Pb(II) 217.0 nm (spectral bandwidth 1.0 nm, lamp current 10 mA) and Cu(II) 324.8 nm (spectral bandwidth 0.5 nm, lamp current 4 mA). Real samples for measurement were prepared using microwave mineralization, according to the method [31].

### 2.9. Descriptive Statistics

Data obtained from the system NOVA were graphically and mathematically processed using Microsoft Excel^®^ and Microsoft PowerPoint^®^. Results were expressed as the mean ± the confidence interval (n = 5, α = 0.05). The detection limits (3 signal/noise, S/N) were calculated according to Long and Winefordner, while N was expressed as a standard deviation of noise determined in the signal domain [32]. The relative standard deviation (RSD%) for repeated measurements at the LOD concentrations for Zn(II), Cd(II), Pb(II), Cu(II) and Ag(I) using the Cr-CPE was less than 9.5% and using the Cr-SPE it was less than 8.9%.

## 3. Results and Discussion

### 3.1. Modification of Carbon Paste with Chromium(III)

**Figure 1:**
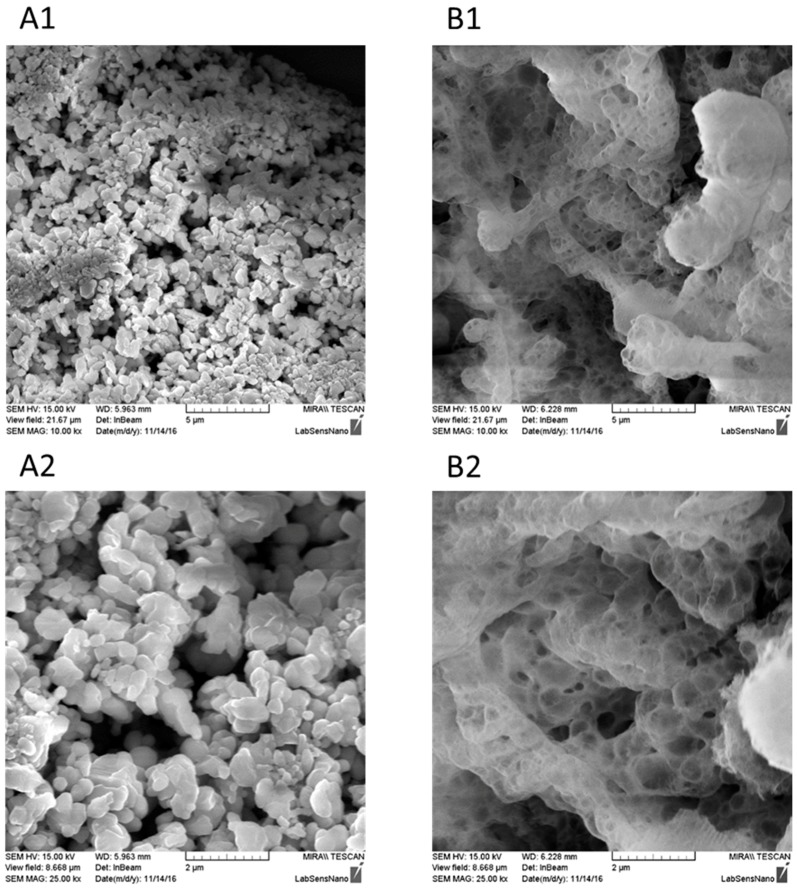
(**A**) Structure of the purchased chromium(III) oxide and (**B**) structure of the thermally produced chromium(III) oxide. Enlargement (**A1**,**B1**) is 15,000×, (**A2**,**B2**) 25,000×.

SEM analysis was used to confirm the porosity of chromium(III) oxide, the first thermally produced from ammonium dichromate or the second commercially purchased. Figure 1 shows the difference in the structure of the purchased chromium(III) oxide (Figure 1(A1,A2)) and the thermally produced one (Figure 1(B1,B2)). The purchased chromium(III) oxide creates a rod-like structure. On the other hand, the structure of the chromium(III) oxide produced by the ammonium dichromate decomposition is uniform and more porous, thus the prepared compound has a larger surface area [3]. According to the BET analysis, the surface area of the purchased chromium(III) oxide was 2.51 m^2^∙g^−1^. The adsorption/desorption isotherm of the thermally produced Cr_2_O_3_ was type III according to IUPAC classification, with hysteresis H3, which corresponds to very weak adsorbate-adsorbent interaction and very large pores. The surface area was 60.3 m^2^∙g^−1^, the total pore volume was 0.245 cm^2^∙g^−1^ and the average pore diameter was 17.16 nm.

**Figure 2:**
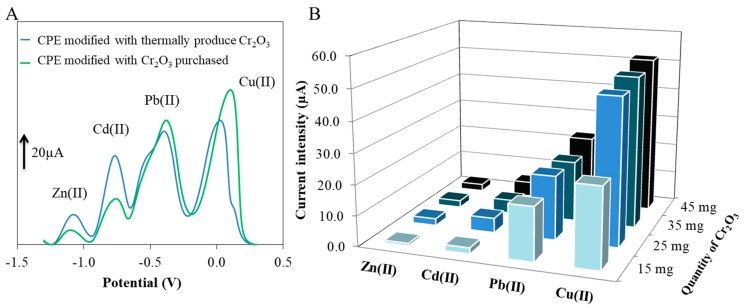
(**A**) Comparison of the influence of the heavy metals detection with the CPE modified with purchased Cr_2_O_3_ compared to CPE modified with thermally produced Cr_2_O_3_. (**B**) The effect of Cr_2_O_3_ addition on detection sensitivity for selected metals.

The structure of both tested compounds was responsible for their differences in affinities for detected metals. In Figure 2A, it is shown that when the purchased chromium(III) oxide was used, a significantly reduced sensitivity of the electrode for the Zn(II) and Cd(II) ions was attained. On the other hand, the sensitivity to lead and copper ions was slightly improved. It is known that the determination of Zn(II) by anodic stripping voltammetry is affected by the presence of Cu(II) [33]. The objective was to achieve a balance of sensitivity for both Cu(II) and Zn(II). This equilibrium was attained using a carbon paste enriched with synthesized chromium(III) oxide, as this material even showed improved composition and sensitivity of the working electrode for Cd(II) ions.

After the optimization of the source for chromium(III) oxide, we turned our attention to the optimization of amounts of the individual carbon paste components. Figure 2B shows the results of this optimization process. Primarily, the ratio of expanded graphite and chromium(III) oxide was optimized. The batch size of the expanded graphite was 0.1 g and then various amounts of chromium(III) oxide (15 mg, 25 mg, 35 mg and/or 45 mg) were added. The same level of response for all metal ions was achieved using 25 mg and 35 mg of chromium(III) oxide. However, when the batch size chromium(III) oxide was increased to 45 mg, an unwanted peak appeared in the voltammogram at the potential of 0.0 V. Therefore, 25 mg of chromium(III) oxide was used in the following experiments. For testing the CPE, the weight ratio of 1:4 was chosen. To achieve the proper consistency in the CPE, 300 µL of paraffin oil was used. The Cr-CPE was also compared with a bare paste electrode (without modification). The results of this comparison are shown in Figure S1.

Finally, the following measurement parameters were optimized as follows (not shown): deposition potential (from −1.5 to −1.3 V), accumulation time (from 60 to 300 s), amplitude (from 60 to 200 mV) and frequency (from 60 to 220 Hz). We found the following to be optimal: deposition potential (−1.3 V), accumulation time (100 s), pulse amplitude (150 mV) and frequency (150 Hz).

The precision of the Cr-CPE was determined by repeatability (same day) and intermediate precision (inter-day). Repeatability was evaluated by analyzing the standard metal solution three times a day. Measurement accuracy was evaluated by comparing the results obtained on three different days. The RSD of the predicted concentrations from the regression equation was taken as precision. For the measured concentration, the relative values of the standard deviation were in the interval and between days ≤9.34%.

The bare electrode, the Cr-CPE and the Cr-SPE behaviour were examined in the range from −1.3 to 0.3 V with CV. The recordings are shown in Figure S2. There are no signals in the case of the bare electrode and the Cr-CPE. For further measurements, the range from −1.6 to 1.6 V was used.

### 3.2. Electrochemical Determination of Individual Zinc, Cadmium, Lead and Copper Ions

Prior to the simultaneous detection of zinc, cadmium, lead and copper ions, calibration curve of each individual metal ion was measured. The equations coefficient of the calibration curves (*I*_p_ = a*c*_m_ + b) and the coefficients of the determination (r^2^) of individual metals are shown in Table 1. A detailed discussion to compare the results achieved by simultaneous detection and detection of individual metal ions can be found in Section 3.3. Graphs of the calibration curves of individual metal ions are given in Figure S3.

### 3.3. Simultaneous Detection of Zinc, Cadmium, Lead, Copper and Silver Ions

**Figure 3:**
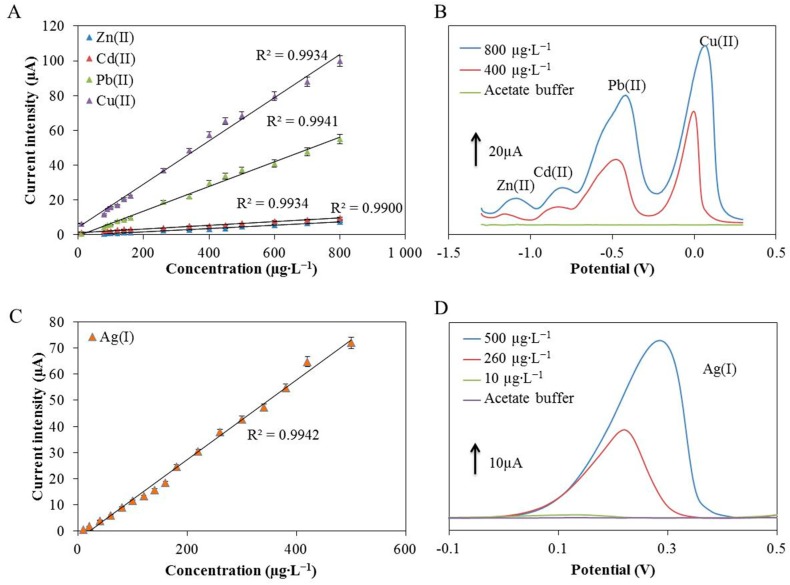
The calibration curves of (**A**) Zn(II), Cd(II), Pb(II) and Cu(II), and (**C**) Ag(I) measured by the carbon paste electrode modified with thermally produced chromium(III) oxide. Typical voltammograms show the simultaneous detection of (**B**) Zn(II), Cd(II), Pb(II), Cu(II), and (**D**) Ag(I), at different concentrations.

The Cr-CPE was optimized for the simultaneous determination of metal ions (Zn(II), Cd(II), Pb(II) and Cu(II)). The calibration curves for each metal are shown in Figure 3A and their coefficients in Table 1, where the values of r^2^ are also presented showing the very good reliability of this method. The error bars were calculated from the standard deviations of the measurements. Typical voltammograms of simultaneous analyses of the metals are shown in Figure 3B. When comparing the detection of individual metal ions and simultaneous ions detection (Table 1), we reached the same LODs, which are shown in Table 2 at the bottom, but their slopes (sensitivities) differ only where an increase in sensitivity for copper and lead ions is noticeable in the case of simultaneous detection. The Cr-CPE is therefore suitable for both types of detection (Table 1, Figure 3A and Figure S1). Moreover, the Zn(II), Cd(II), Pb(II) and Cu(II) detection performance of the proposed sensor was compared with other previously reported modified carbon paste electrodes and the results are listed in Table 2. It clearly follows from the results obtained in this study that our electrodes have comparable analytical accuracy to other electrodes published previously.

Besides the simultaneous detection of the mentioned metals, we also tested the developed sensor to determine Ag(I). Prior to the measurement of the calibration curve of Ag(I) ions, optimization of the selected experimental parameters was carried out. However, it was found that the most suitable parameters were the same as in the case of simultaneous detection of Zn(II), Cd(II), Pb(II) and Cu(II) ions. It can be concluded that the CPE modified with chromium(III) oxide can detect five metals at low concentrations in a single mode measurement. As in the previous measurement, an acetate buffer of pH 5 was used for the detection of silver ions. As it is shown in Figure 3C, silver ions can be measured within the linear range of 10 to 500 µg·L^−1^. Ag(I) was characterized by the equation *I*_p_ = 0.1525*c*_m_ − 3.1774 with the coefficient of determination of 0.9942. The limit of detection was determined to be 3 µg·L^−1^. Typical voltammograms of analyses of Ag(I) are shown in Figure 3D.

Table 3 contains a comparison of the Cr-CPE with previous studies on the electroanalytical detection of Ag(I). Some mentioned methods achieve lower detection limits of silver cations on the surface of the modified electrode, having accumulation times which are several times longer than our time of 100 s. A shorter accumulation time enables faster measurements, therefore in the same time more analyses can be made. However, a great advantage of our modification is the simple and easily reproducible manufacturing and low manufacturing cost.

### 3.4. Interferences

**Figure 4:**
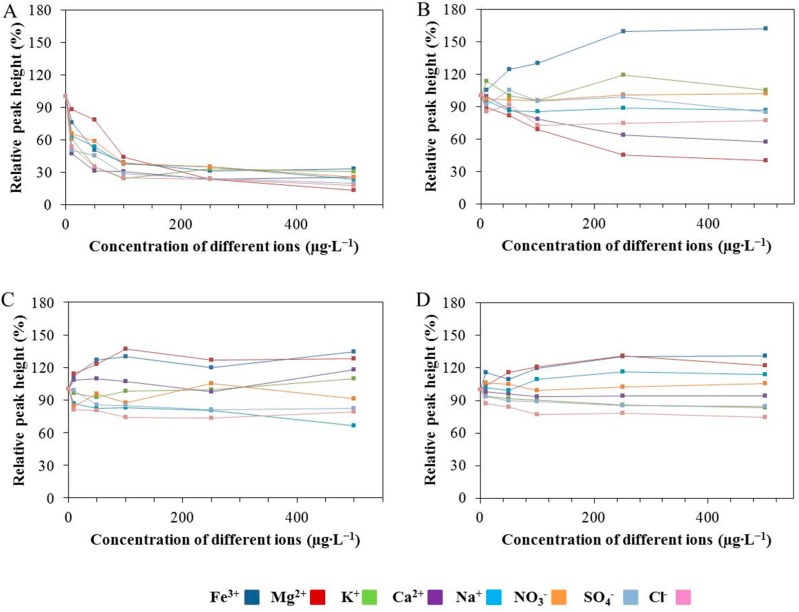
(**A**) The effect of different ions (Fe^3+^, Mg^2+^, K^+^, Ca^2+^, Na^+^, NO_3_^−^, SO_4_^2−^, Cl^−^) on the relative peak height of Zn(II), (**B**) Cd(II), (**C**) Pb(II) and (**D**) Cu(II) measured by the Cr-CPE in a mixture of Zn(II), Cd(II), Pb(II) and Cu(II). The concentration of each component in the mixture was 140 µg∙L^−1^.

In this part of the study, the response of the chromium(III) oxide-modified CPE to various interferences in the mixture was tested. Different ions (Fe^3+^, Mg^2+^, K^+^, Ca^2+^, Na^+^, NO_3_^−^, SO_4_^2−^, Cl^−^ in the form of Fe(NO_3_)_3_, Mg(NO_3_)_2_, KNO_3_, Ca(NO_3_)_2_, NaNO_3_, HNO_3_, H_2_SO_4_ and HCl) were added to the mixture of Zn(II), Cd(II), Pb(II) and Cu(II) and the specific signals of Zn(II), Cd(II), Pb(II) and Cu(II) were observed. The results are shown in Figure 4A–D. The first point of the curve represents values free from the influence of interferences. The influence of these ions on the relative peak height of Zn(II) is shown in Figure 4A, where it can be seen that with the addition of each ion, the sensitivity for Zn(II) decreases. Figure 4B shows the influence of different ions on the relative peak height of cadmium. It follows from the results obtained that the signal of Cd(II) increases with the addition of ferric ions and conversely this signal decreases with the addition of magnesium ions. The relative peak height of lead increases with the amount of ferric and magnesium ions and decreases with chloride ions (Figure 4C). Figure 4D shows the dependence of the Cu(II) relative peak height on the concentrations of different salts. It can be seen that the Cu(II) signal increases with the increasing concentration of ferric and magnesium ions. On the other hand, this signal decreases in correlation to the increase of the chloride ions concentration. The response of Cu(II) ions to interference has similar characteristics to the case of Pb(II). It can be summarized that with the increasing concentration of ferric ions the relative peak heights of Cd(II), Pb(II) and Cu(II) increase. The same effect can also be observed in the case of magnesium salts but for Pb(II) and Cu(II) ions only. In contrast, in presence of magnesium ions the response of cadmium ions achieves a markedly lowered value than expected. On the other hand, different concentrations of KNO_3_, Ca(NO_3_)_2_ and HNO_3_ did not have any influence on the Cd(II), Pb(II) and Cu(II).

**Figure 5:**
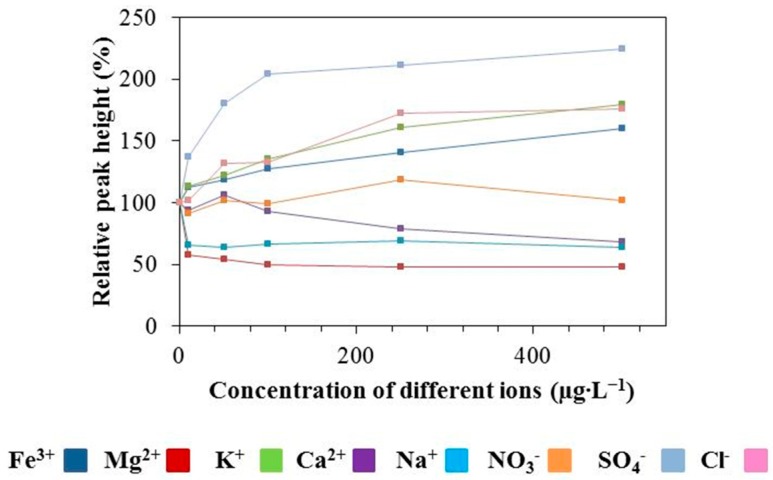
The effect of different ions (Fe^3+^, Mg^2+^, K^+^, Ca^2+^, Na^+^, NO_3_^−^, SO_4_^2−^, Cl^−^) on the relative peak height of Ag(I) measured by the Cr-CPE in a mixture of Ag(I). The concentration of Ag(I) was 140 µg∙L^−1^.

As in the case of simultaneous detection, the influence of different types of anions and cations on silver determination was studied. In this part of the experiment, it was necessary to realize that in the case of chloride ions, it was important not to exceed the solubility equilibrium. If the value of the solubility equilibrium was exceeded, precipitation of silver chloride from the mixture would occur. For AgCl this value is equal to 1.8 × 10^−10^. Considering this fact, the same types of cations and anions as in the case of the simultaneous analyses of the abovementioned metals have been tested (see above). Figure 5 shows that the increasing SO_4_^2−^ concentration led to the increase of the corresponding relative peak height of silver. In contrast, the addition of Mg^2+^ and Na^+^ decreased the silver signal. The response of the silver ions was more stable in the presence of NO_3_^−^.

### 3.5. The Chromium Modified Screen-Printed Electrode (Cr-SPE)

**Figure 6:**
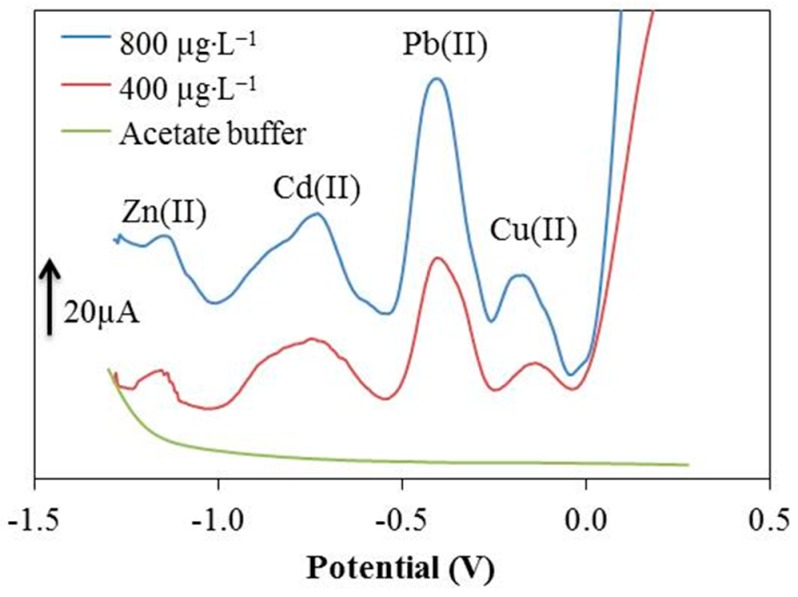
Typical voltammograms of simultaneous detection of Zn(II), Cd(II), Pb(II) and Cu(II) on the Cr-SPE at different concentrations.

The carbon based paste modified with chromium(III) oxide was used for printing of sensors. For comparison, the sensitivity was measured within the same range of concentrations under the same optimal parameters as in the case of the Cr-CPE. The Cr-SPE sensor, based on silver collectors, means it is not suitable to directly detect Ag(I). We therefore tested the Cr-SPE sensors without the silver collectors. The results showed poor stability and a very high limit of detection for all tested metal ions. Therefore, other experiments were performed using the Cr-SPE with silver collectors for the detection of Zn(II), Cd(II), Pb(II) and Cu(II). A comparison of the analytical parameters of the Cr-CPE and the Cr-SPE is shown in Table 4. Figure 6 shows the comparison of results obtained from paste and printed sensors. Since the production of these sensors is relatively inexpensive and the Cr-SPEs show good detection limits for the simultaneous determination of selected metals, it could be used in the future for practical application. Another great advantage is the speed, precision and high reproducibility.

### 3.6. Analysis of Real Samples

The Cr-SPE functionality was verified on real samples of industrial wastewater. The sample was taken from a chemical factory in the Czech Republic. Results obtained by this method were compared with those obtained by AAS. The determined concentrations of Zn(II), Cd(II), Pb(II) and Cu(II) based on the present Cr-SPE method using Cr-SPE are presented in Table 5. Ag(I) ions have not been detected. It clearly follows from the results obtained that there is a consensus confirming the application potential of the developed Cr-SPE.

## 4. Conclusions

Simultaneous analysis of Zn(II), Cd(II), Pb(II) and Cu(II) was successfully performed using the Cr-CPE. This modification of the electrode showed good stability and high sensitivity. The sensitivity of such prepared electrodes is satisfactory in comparison with other methods (see Section 3.2, Table 2 and Table 3). One may suggest that the main advantages of our proposed system are simplicity, speed, very good repeatability and low cost of preparation. Moreover, it offers a good alternative where electrochemical measurements using mercury electrodes cannot be used. In addition, it is possible to successfully detect Ag(I) ions with a detection limit of 3 µg·L^−1^ using the Cr-CPE. A practical application for this Cr-CPE is its successful transfer to the printed sensors, also used in this study. Cr-SPEs are very sensitive especially in the case of Pb(II) and Cu(II). The same detection limits as the Cr-CPE were achieved, however the Cr-SPE is useful for the simultaneous detection of all four investigated metals (Zn(II), Cd(II), Pb(II) and Cu(II)). Furthermore, the Cr-SPE proved to be a useful tool for the detection of metals in practice, where the estimated detection limits comply with FAO (Food and Agriculture Organization) and WHO (World Health Organization) recommended maximum concentrations of trace elements in water irrigation and livestock drinking water [46]. Therefore, these sensors can be put to practical use in portable field detection devices.

## Figures and Tables

**Table 1:** The equation coefficients (*I*_p_ = a*c*_m_ + b) and the coefficient of determination (r^2^) for each individual heavy metal ion (Zn(II), Cd(II), Pb(II), and Cu(II)) and for the mixture solution determined by the Cr-CPE.

Electrode Type	Detected Ion	a	b	r^2^
Cr-CPE	Zn(II)	0.0109	−0.4783	0.9905
(individual ions)	Cd(II)	0.0177	1.5649	0.9890
	Pb(II)	0.0566	−0.6693	0.9952
	Cu(II)	0.1104	0.4032	0.9904
Cr-CPE	Zn(II)	0.0096	−0.4081	0.9900
(mixture solution)	Cd(II)	0.0103	1.1684	0.9934
	Pb(II)	0.0710	0.7963	0.9941
	Cu(II)	0.1244	3.9573	0.9934

**Table 2:** Comparison of the performance of the proposed electrode with other modified carbon paste electrodes for simultaneous detection of heavy metals.

Electrode Type	Detected Metal	Analysis Method	LOD (µg∙L^−1^)	Linear Range (µg∙L^−1^)	Accumulation Time (s)	References
BRMCPE ^1^	Zn(II)	SWASV	134	400–1000	300	[34]
	Cd(II)		155	400–1000		
	Pb(II)		15	50–200		
	Cu(II)		125	250–700		
HMS-Qu/CPE ^2^	Cd(II)	DPV ^7^	0.1	0.5–229	120	[35]
	Pb(II)		0.2	2–1658		
	Cu(II)		0.3	1–381		
N-BDMP ^3^	Cd(II)	SWASV	7	10–2000	210	[36]
	Hg(II)		8	10–2000		
Ac-Phos SAMMS ^4^	Cd(II)	SWASV	0.5	10–200	1200	[37]
	Cu(II)		0.5	10–200		
	Pb(II)		0.5	10–200		
MWCNT/CPE ^5^	Zn(II)	PSA ^8^	28	58–646	180	[18]
	Cd(II)		8	58–646		
	Pb(II)		7	58–646		
OPFP with bismuth film ^6^	Pb(II)	SWASV	0.1	1–100	120	[38]
	Cd(II)		0.1	1–100		
Cr-CPE	Zn(II)	SWASV	25	80–800	100	This work
	Cd(II)		3	10–800		
	Pb(II)		3	10–800		
	Cu(II)		3	10–800		

^1^ BRMCPE—black rice modified carbon paste electrode; ^2^ HMS-Qu/CPE—hexagonal mesoporous silica immobilized quercetin carbon paste electrode; ^3^ N-BDMP—phosphorous ylide nitro benzoyl diphenylmethylenphosphorane carbon paste electrode; ^4^ Ac-Phos SAMMS—carbon paste electrode modified with carbamoylphosphonic acid self-assembled monolayer on mesoporous silica; ^5^ MWCNT/CPE—multiwalled carbon nanotube electrode; ^6^ OPFP with bismuth film—ionic liquid n-octylpyridinium hexafluorophosphate modified carbon paste electrode with bismuth film; ^7^ DPV—differential pulse voltammetry; ^8^ PSA—potentiometric stripping analysis.

**Table 3:** Comparison of the performance of the proposed electrode with other reported electrochemical silver sensors.

Electrode Type	Analysis Method	LOD (µg∙L^−1^)	Linear Range (µg∙L^−1^)	Accumulation Time (s)	References
MGCE modified with Fe_3_O_4_-Au NPs ^1^	DPV	6	13–1910	300	[39]
CPE modified with AMQ ^2^	DPASV ^8^	0.4	0.9–302	720	[40]
CPE modified with GSN-TH-DPA ^3^	POT ^9^	0.5	0.9–1079000	-	[41]
CPE modified with IIP ^4^	DPSV ^10^	0.1	0.3–92	360	[42]
CPE modified with PAR ^5^	DPASV	0.1	0.5–302	720	[43]
CPE modified with IIP-MWCNTs	DPSV	0.01	0.05–30	180	[4]
CPE modified with NBHAE-MWCNTs ^6^	DPASV	0.09	0.5–194	540	[44]
CPE modified with DPSG ^7^	POT	11	54–10790000	-	[45]
Cr-CPE	SWASV	3	10–500	100	This work

^1^ MGCE modified Fe_3_O_4_-Au NPs—Magnetic glassy carbon electrode modified iron oxide—gold nanoparticles; ^2^ CPE modified with AMQ—3-Amino-2-mercapto quinazolin-4(3H)-one; ^3^ CPE modified with GSN-TH-DPA—Graphene nanosheets–thionine–diphenylacetylene, ^4^ CPE modified with IIP—Ion imprinted polymer–poly(vinyl chloride); ^5^ CPE modified with PAR—4-(2-pyridylazo)-resorcinol; ^6^ CPE modified with NBHAE-MWCNTs—N,N´-bis(2-hydroxybenzylidene)-2,2´(aminophenylthio)ethane; ^7^ CPE modified with DPSG—dipyridyl-functionalized silica gel; ^8^ DPASV—differential pulse anodic stripping voltammetry; ^9^ POT—potentiometry; ^10^ DPSV—differential pulse stripping voltammetry.

**Table 4:** Comparison of the results on the Cr-CPE and Cr-SPE for simultaneous detection of Zn(II), Cd(II), Pb(II) and Cu(II) in mixture solution.

Electrode Type	Detected Ion	LOD (µg∙L^−1^)	Linear Range (µg∙L^−1^)
Cr-CPE	Zn(II)	25	80–800
	Cd(II)	3	10–800
	Pb(II)	3	10–800
	Cu(II)	3	10–800
Cr-SPE	Zn(II)	350	400–800
	Cd(II)	25	80–800
	Pb(II)	3	10–800
	Cu(II)	3	10–800

**Table 5:** Comparison of Cr-SPE, HMDE and AAS for the determination of Zn(II), Cd(II), Pb(II) and Cu(II) in real wastewater samples.

Type of Measurement	Zn(II) (mg∙L^−1^)	Cd(II) (mg∙L^−1^)	Pb(II) (mg∙L^−1^)	Cu(II) (mg∙L^−1^)
Cr-SPE	2.6 ± 0.8	3.5 ± 0.7	5.7 ± 1.1	7.9 ± 0.6
HMDE ^1^	6.2 ± 0.5	3.9 ± 0.3	4.7 ± 0.3	8.3 ± 0.5
AAS	6.6 ± 0.01	4.2 ± 0.04	4.8 ± 0.02	8.8 ± 0.01

^1^ HMDE—hanging mercury drop electrode.

## Supplementary Materials

The following are available online at http://www.mdpi.com/1424-8220/17/8/1832/s1, Figure S1: Comparison of the influence of the heavy metals detection with the bare electrode compared to the CPE modified with thermally produced Cr_2_O_3_. The concentration of individual metals was 800 µg∙L^−1^; Figure S2: Cyclic voltammetry of individual materials. (**a**) CV for the bare electrode, the range from −1.3 to 0.3 V, (**b**) CV for the bare electrode, the range from −1.6 to 1.6 V, (**c**) CV for the Cr-CPE, the range from −1.3 to 0.3 V, (**d**) CV for the Cr-CPE, the range from −1.6 to 1.6 V, (**e**) CV for the Cr-SPE, the range from −1.3 to 0.3 V, (**f**) CV for the Cr-SPE, the range from −1.6 to 1.6 V; Figure S3: The calibration curves for each metals ions Zn(II), Cd(II), Pb(II) and Cu(II), measurement by the Cr-CPE.
