# Supplementary material for: Determination of Zinc, Cadmium, Lead, Copper and Silver Using a Carbon Paste Electrode and a Screen Printed Electrode Modified with Chromium(III) Oxide
Source: Sensors (Basel). 2017 Aug 9;17(8):1832. doi: 10.3390/s17081832 (PMC5580028; doi:10.3390/s17081832)
Supplement: Supplementary file 1 [file sensors-17-01832-s001.pdf]

Determination of Zinc, Cadmium, Lead, Copper and Silver Using a Carbon Paste Electrode and a Screen Printed Electrode Modified with Chromium(III) Oxide

Zuzana Koudelkova [1], Tomas Syrovy [2,3], Pavlina Ambrozova [4], Zdenek Moravec [5], Lubomir Kubac [3,6], David Hynek [1,7], Lukas Richtera [1,7], Vojtech Adam [1,7,*]

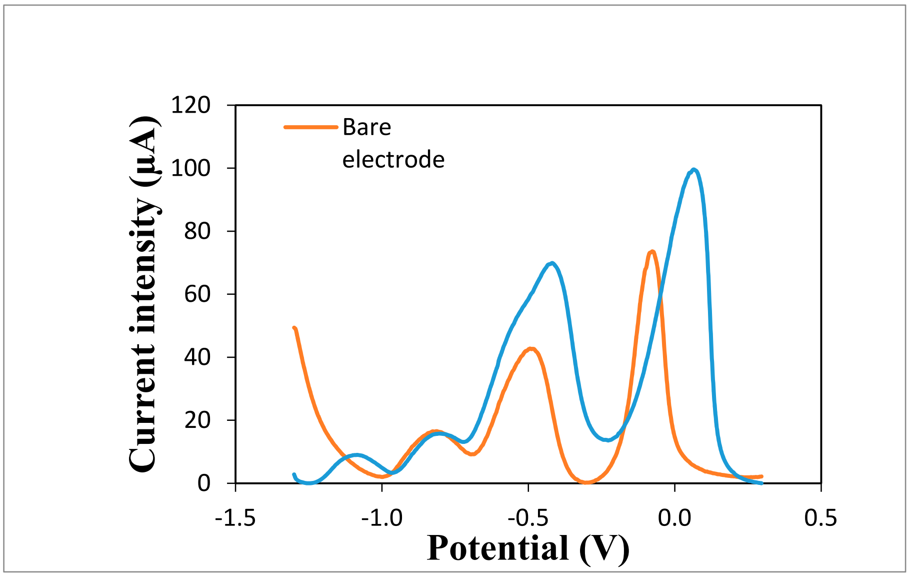

Figure S1. Comparison of the influence of the heavy metals detection with the bare electrode compared to the CPE modified with thermally produced Cr_2O_3. The concentration of individual metals was 800 µg·L^{-1}.

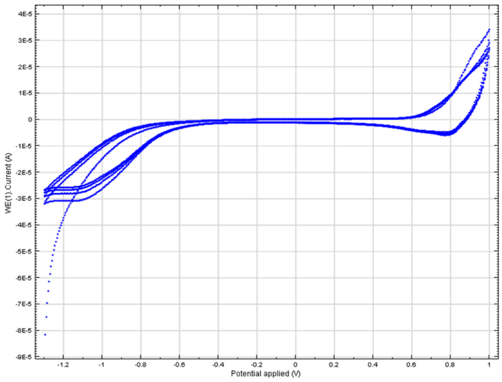

(**a**)

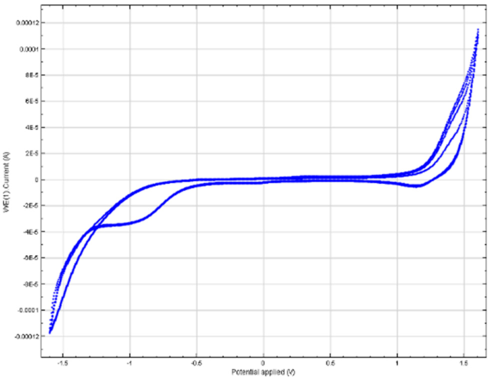

(**b**)

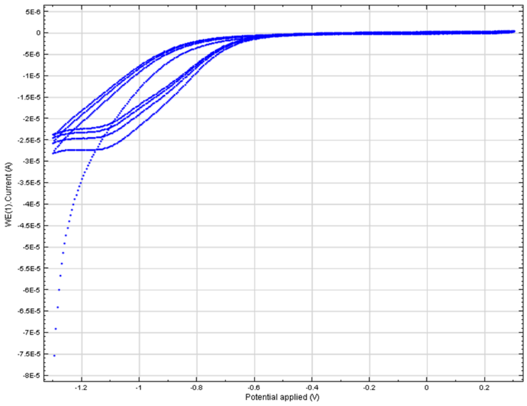

(**c**)

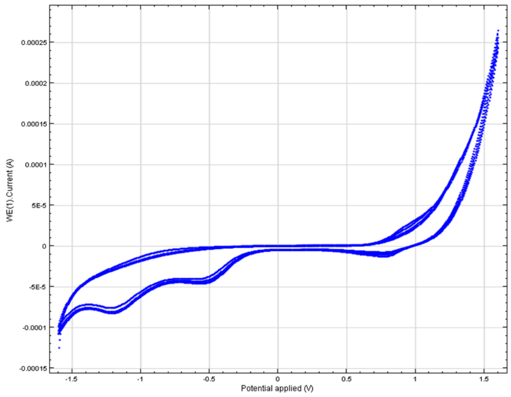

(**d**)

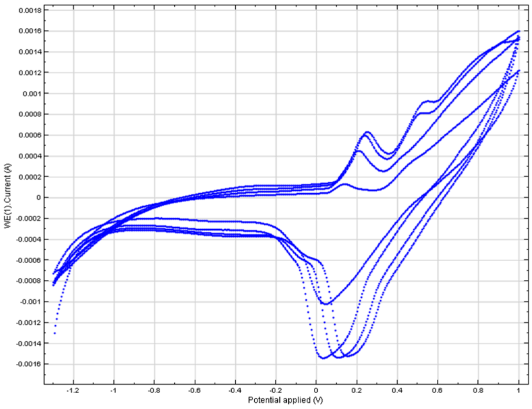

(**e**)

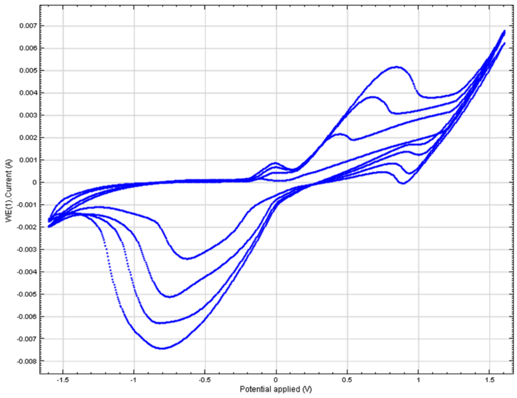

(**f**)

Figure S2. Cyclic voltammetry of individual materials. (**a**) CV for the bare electrode, the range from −1.3 to 0.3 V, (**b**) CV for the bare electrode, the range from −1.6 to 1.6 V, (**c**) CV for the Cr-CPE, the range from −1.3 to 0.3 V, (**d**) CV for the Cr-CPE, the range from −1.6 to 1.6 V, (**e**) CV for the Cr-SPE, the range from −1.3 to 0.3 V, (**f**) CV for the Cr-SPE, the range from −1.6 to 1.6 V.

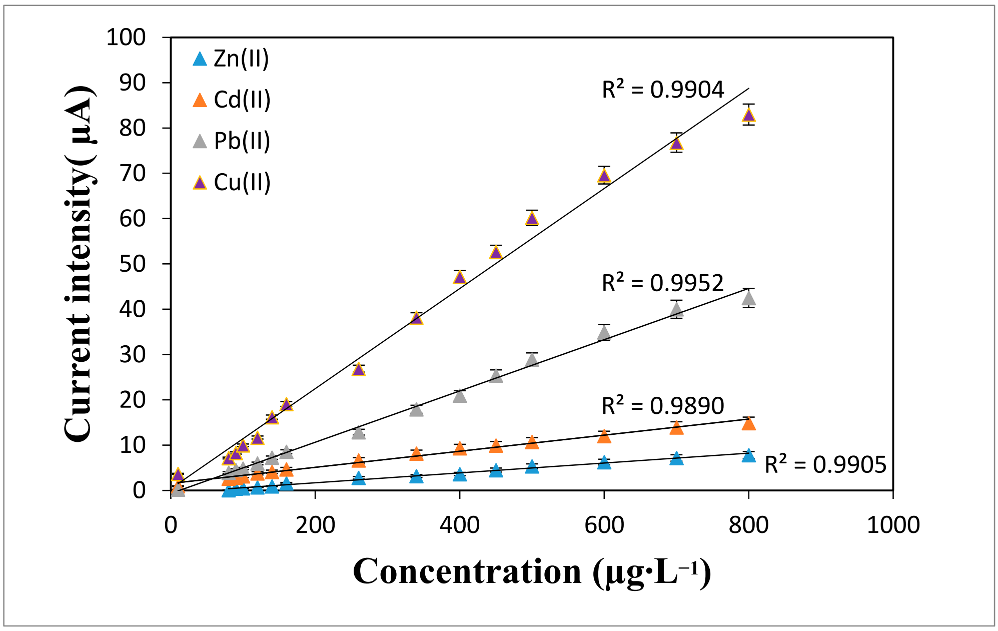

Figure S3. The calibration curves for each metals ions Zn(II), Cd(II), Pb(II) and Cu(II), measurement by the Cr-CPE.
